# Procalcitonin as a prognostic factor in patients with acute exacerbation of COPD without pneumonia: a prospective multicenter cohort study

**DOI:** 10.3389/fmed.2026.1905875

**Published:** 2026-10-20

**Authors:** Min Qi, Chen Zhou, Yuanming Luo, Hailong Wei, Huiqing Ge, Huiguo Liu, Jianchu Zhang, Xianhua Li, Pinhua Pan, Xiufang Xie, Mengqiu Yi, Lina Cheng, Jiarui Zhang, Lige Peng, Jiaqi Pu, Xueqing Chen, Haixia Zhou, Yongjiang Tang, Qun Yi

**Affiliations:** 1 Department of Respiratory and Critical Care Medicine, West China Hospital, Sichuan University, Chengdu, Sichuan, China; 2 Department of Geriatric Intensive Care Unit, Sichuan Provincial People’s Hospital, University of Electronic Science and Technology of China, Chengdu, China; 3 Center of Infectious Diseases, Division of Infectious Diseases in State Key Laboratory of Biotherapy, West China Hospital, Sichuan University, Chengdu, Sichuan, China; 4 State Key Laboratory of Respiratory Disease, Guangzhou Medical University, Guangzhou, Guangdong Province, China; 5 Department of Respiratory and Critical Care Medicine, People’s Hospital of Leshan, Leshan, Sichuan, China; 6 Department of Respiratory and Critical Care Medicine, Sir Run Run Shaw Hospital, Zhejiang University School of Medicine, Hangzhou, Zhejiang, China; 7 Department of Respiratory and Critical Care Medicine, Tongji Hospital, Tongji Medical College, Huazhong University of Science and Technology, Wuhan, Hubei, China; 8 Department of Respiratory and Critical Care Medicine, Union Hospital, Tongji Medical College, Huazhong University of Science and Technology, Wuhan, Hubei, China; 9 Department of Respiratory and Critical Care Medicine, The First People’s Hospital of Neijiang City, Neijiang, Sichuan, China; 10 Department of Respiratory and Critical Care Medicine, Xiangya Hospital, Central South University, Changsha, Hunan, China; 11 Department of Emergency, First People's Hospital of Jiujiang, Jiu jiang, Jiangxi Province, China; 12 Sichuan Cancer Hospital, University of Electronic Science and Technology of China, Chengdu, Sichuan, China

**Keywords:** AECOPD, inflammatory biomarker, mortality, procalcitonin, prognostic factors

## Abstract

**Background/objectives:**

Pneumonia frequently precipitates AECOPD, and procalcitonin (PCT) has been utilized to guide antibiotic administration in these patients. However, the specific role and clinical significance of PCT in AECOPD patients who do not have pneumonia remains unexplored and is yet to be thoroughly evaluated.

**Methods:**

We conducted univariate and multivariate cox regression analyses to identify independent risk factors for in-hospital mortality among AECOPD patients. The Youden index was applied to determine the optimal cutoff value for PCT, which was then used to categorize participants into two groups based on this threshold. Subsequently, we compared the clinical manifestations and outcomes of patients between these groups, and Kaplan–Meier curves were con-structed and analyzed using the log-rank test. A subgroup analysis was also conducted in patients with/without antibiotic using during hospitalization.

**Results:**

A total of 6,791 hospitalized AECOPD patients of 10 tertiary care hospitals within China was enrolled in our study from September 2017 to July 2021. Univariable and multivariable cox regression analyses revealed that PCT (HR 1.016, 95% CI 1.003–1.029, *p* = 0.014) as independent risk factors for in-hospital mortality in AECOPD patients. The ROC analysis established an optimal cut-off value of 0.11 for predicting in-hospital mortality. The PCT high group (≥0.11 ng/mL) demonstrated higher proportion of chronic underlying diseases, and higher age-adjusted Charlson comorbidity index score, increased in-hospital mortality, greater ICU admission rates, higher rates of invasive mechanical ventilation, and a longer length of stay (all *P* < 0.001). PCT and the cut-off value of 0.11 could also have effective prognostic value in both subgroups of patients with/without antibiotic usage.

**Conclusions:**

PCT emerged as an independent risk factor for in-hospital mortality in AECOPD patients without pneumonia. Those with elevated PCT levels (≥0.11 ng/mL) exhibited a higher burden of comorbidities, a potential of pro-infection or pro-inflammation and poorer clinical outcomes.

**Clinical Trial Registration:**

http://www.chictr.org.cn/showproj.aspx?proj=12162, identifier ChiCTR2100044625.

## 1 Introduction

Chronic obstructive pulmonary disease (COPD) is a heterogeneous lung disease characterized by persistent airflow limitation (1) and is the leading cause of global mortality and disability (2, 3). Acute exacerbation of COPD (AECOPD), the primary cause of death in patients with COPD, always involves hospitalization, adverse outcomes and a decline in their quality of life and death (4, 5). As a result, identifying predictors of poor in-hospital outcome is a clinical priority in AECOPD inpatients.

Respiratory infections—predominantly bacterial—represent the most important and prevalent trigger of AECOPD, accounting for approximately 50%–70% of hospitalized exacerbation episodes (1, 6, 7). As a result, CRP and neutrophil–lymphocyte ratio (NLR) has been identified as the predictors of clinical outcomes based on the inflammation status of AECOPD patients (8, 9). Nevertheless, beyond overt respiratory infection, AECOPD patients are immunocompromised under the condition of systemic corticosteroid use, malnutrition, and impaired mucociliary clearance. Then, potential extrapulmonary infection or tissue injuries might appear, which may also affect their prognosis. As a result, although inflammation biomarkers are prognostic factors, they still fail to comprehensively uncover the subclinical infection or tissue injuries in these patients.

Procalcitonin (PCT), the precursor of calcitonin, is typically produced by the C cells of the thyroid gland and is barely detectable in the serum under normal physiological conditions (10). It is increasingly recognized that PCT is synthesized in mononuclear cells and the liver, with lipopolysaccharides and cytokines acting as primary stimulants for its production (11). In contrast to non-specific inflammatory markers such as C-reactive protein (CRP), PCT has emerged as a pivotal biomarker for systematic bacterial infections, guiding the clinical decision-making process regarding antibiotic use (10, 12, 13). In AECOPD specifically, PCT has been validated for differentiating bacterially triggered from non-bacterial exacerbations and for guiding antibiotic prescription, thereby reducing antibiotic overuse and mortality risks (14, 15). Also, elevated PCT (>0.25 ng/mL) was reported to be significantly associated with poor prognosis in AECOPD patients (16, 17), which was related to bacterial infection. Beyond this infection-driven role, however, accumulating evidence suggests that PCT elevation may also reflect pathophysiological processes unrelated to overt bacterial infection.

Beyond its established role in infectious disease diagnosis, PCT has also been shown to carry prognostic significance in several non-infectious or mixed clinical settings. For instance, elevated pre-treatment PCT level has been identified as a predictor of progression-free survival in small-cell lung cancer (18). Also, postoperative PCT has been identified as a risk stratification tool for delayed complications after cardiac surgery, while CRP could hardly do the prediction (19). Furthermore, elevated PCT, not caused by infectious factors, was reported to serve as an indicator of burn severity, late sepsis and mortality during early burn injury (20). These evidences indicated that PCT is not merely an inflammatory biomarker regarding bacterial infection, but also relates to internal tissue injuries in patients. These observations suggest that PCT may integrate multiple pathophysiological signals—including systemic bacterial invasion, immune dysregulation, and organ stress—that collectively determine patient prognosis. Nevertheless, whether AECOPD patients without confirmed pneumonia or sepsis who nevertheless present with elevated PCT levels is underappreciated. Also, even in AECOPD patients without diagnosed pneumonia or sepsis, the elevation of PCT might also indicate some other pathological issues and then give link to patients’ outcomes. However, the existing evidence regarding PCT’s role as a prognostic marker in AECOPD outcomes for those without pneumonia remains limited.

In this study, we sought to address whether elevated PCT was still observed in AECOPD inpatients without pneumonia or sepsis and its predictive effect for in-hospital mortality in a large, prospective, multicenter cohort. We further leveraged the Youden index to determine the cut-off point for PCT concentrations and validate the prediction efficacy of PCT. Then, the pattern of PCT elevation and its relationship with conventional infectious and inflammatory markers including CRP, WBC, and renal function indices were investigated to provide evidence for or against an occult or extrapulmonary infectious etiology.

## 2 Materials and methods

### 2.1 Patients and study design

Participants for our study were selected from the MAGNET AECOPD Registry (MAnaGement aNd advErse ouTcomes in in-patients with acute exacerbation of COPD), a prospectively multicenter cohort study in China, as identified by the registration number ChiCTR2100044625. Spanning from September 2017 to July 2021, this study prospectively enrolled hospitalized AECOPD patients across a network of 10 prominent tertiary care hospitals within China. The overarching goals of this registry were to scrutinize the management protocols and to survey the spectrum of adverse outcomes—including but not limited to mortality, ICU admissions, invasive mechanical ventilation requirements, and hospital readmissions—among patients hospitalized for AECOPD (21). The specific aim of our investigation was to establish the prognostic significance of PCT in forecasting the outcomes for AECOPD inpatients.

Eligible patients were adults aged 40 years or older admitted with a physician diagnosis of AECOPD according to the GOLD criteria, with a serum PCT measurement available during the index admission. Patients were excluded if: 1) PCT was not measured (*n* = 4,645); 2) they were younger than 40 years (*n* = 40); 3) they had a malignant tumor (*n* = 505); 4) pneumonia was diagnosed (*n* = 1,993); 5) sepsis was diagnosed (*n* = 33). 2) Pneumonia and sepsis were recorded in the case report form as physician-assigned diagnosis made during the routine clinical care at each participating center. The diagnosis of pneumonia was compatible signs and symptoms with radiographic confirmation, in line with the Chinese and international community-acquired pneumonia guidelines (21–23), and the diagnosis of sepsis was based on the Sepsis-3 consensus definition (24). Chest imaging and microbiological sampling were performed at the discretion of the treating team according to routine practice at each center rather than by study protocol.

Ethical approval for this study was granted by the Ethics Committee of our hospital (2019-1056) and other nine academic medical centers involved. Informed consent was obtained from each participant, and the study was performed in compliance with the ethical standards set forth by the Declaration of Helsinki.

### 2.2 Data collection

In the conduct of this study, we systematically gathered the clinical data of the participants utilizing a standardized case report form. This comprehensive data collection included a spectrum of parameters such as baseline demographic characteristics, co-existing medical conditions, symptoms and vital signs at the time of admission, results from laboratory and radiological investigations, and the treatments administered, as previously detailed (21). All the blood tests were collected within 24 h after admission. The age-adjusted Charlson comorbidity index (aCCI) score, a well-established predictive tool for mortality risk assessment based on the presence of co-morbidities (22), was employed to quantify the severity of co-morbid conditions.

### 2.3 Clinical outcomes

In our analysis, we meticulously assessed a spectrum of in-hospital outcomes, encompassing all-cause in-hospital mortality, the need for ICU admission, the utilization of invasive mechanical ventilation, and the duration of hospital stays, denoted as length of stay (LOS).

### 2.4 Statistics

All statistics were analyzed using the R software, version 4.2.2 (https://www.r-project.org). Categorical variables were presented as counts with percentages, while non-normally and normally distributed continuous variables were expressed as medians with interquartile ranges (IQRs) or mean with standard deviations (SDs). For the comparison of continuous variables across two groups, we applied the t-test or the Wilcoxon rank sum test for normally distributed variables and nonnormally distributed variables, respectively. Categorical variables were analyzed using the Chi-square test or Fisher’s exact test as appropriate. Utilizing univariate and multivariate Cox regression models, we discerned factors that were predictive of in-hospital mortality. The receiver operating characteristic (ROC) curve analysis was then employed to identify the most effective PCT cut-off value for forecasting in-hospital mortality. Participants were subsequently divided into two groups based on the PCT cut-off value—those with elevated PCT levels (PCT high) and those with lower levels (PCT low)—and a comparative analysis of their clinical features was conducted. Survival analyses were depicted using Kaplan–Meier curves to represent the survival profiles of these groups. Then, a subgroup analysis was performed in patients treated with/without antibiotics during hospitalization. The predictive value of PCT and the effective cut-off value of PCT in each subgroup was also valued by ROC curve. Additionally, the correlation between PCT levels and a suite of laboratory indices, as well as the aCCI score, was explored using Pearson’s correlation analysis. All statistical evaluations were conducted with a two-tailed approach, considering a *p*-value of less than 0.05 as significant.

## 3 Results

### 3.1 Study population

In the registry study encompassing 14,007 patients with AECOPD, a series of exclusion criteria were applied to refine the study cohort. Specifically, we excluded patients with incomplete PCT data (*n* = 4645), those under the age of 40 (*n* = 40), individuals with malignant tumors (*n* = 505), patients with pneumonia (*n* = 1993), and those diagnosed with sepsis (*n* = 33). Following these exclusions, a total of 6,791 participants remained and were incorporated into the subsequent analysis, as illustrated in Figure 1.

**Figure 1:**
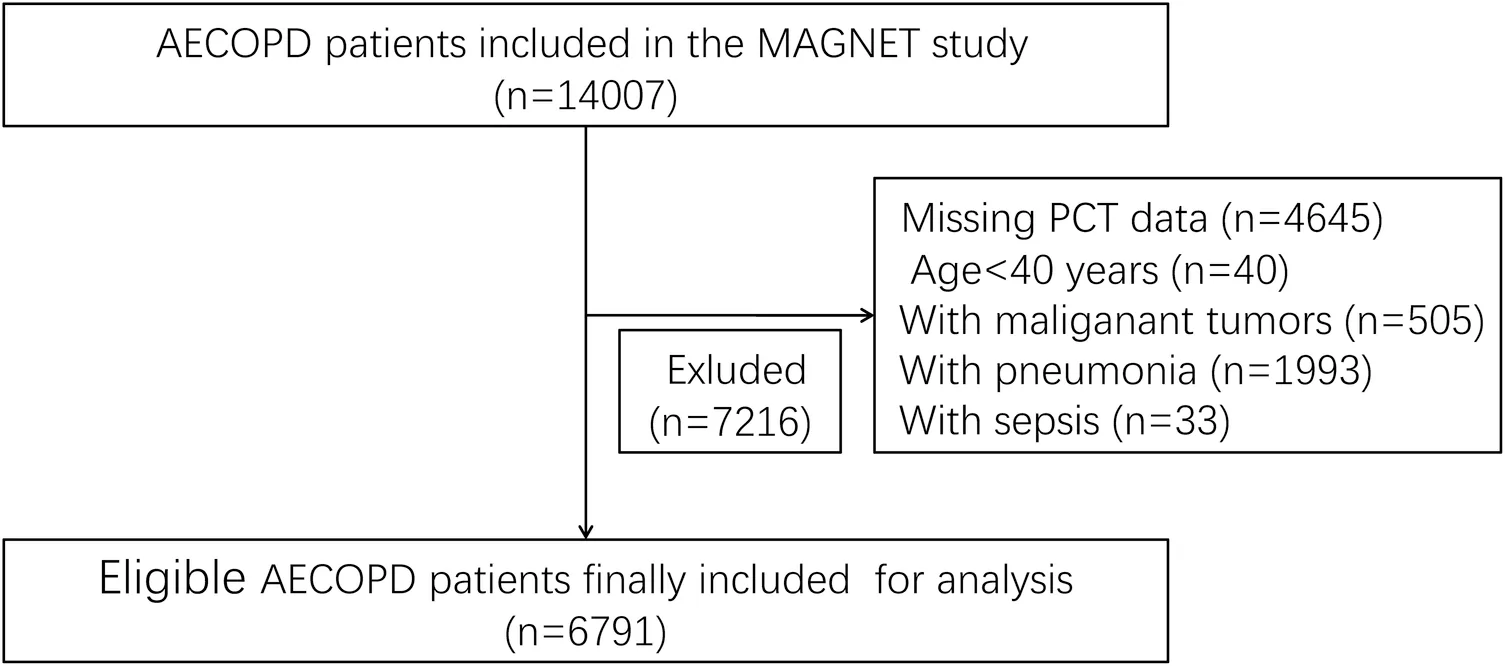
The flowchart of patient enrollment. AECOPD, acute exacerbation of chronic obstructive pulmonary disease; PCT, procalcitonin.

### 3.2 Predictors of in-hospital mortality

We conducted both univariable and multivariable Cox regression analysis to evaluate the baseline characteristics, comorbidity and laboratory tests for in-hospital mortality (Table 1). Notably, these included increasing age (HR 1.032, 95% CI 1.011–1.053, *p* = 0.002), the presence of interstitial lung disease (ILD) (HR 2.036, 95% CI 1.071–3.870, *p* = 0.030), cor pulmonale (HR 1.912, 95% CI 1.276–2.866, *p* = 0.002), diastolic blood pressure (DBP) (HR 0.971, 95% CI 0.954–0.990, *p* = 0.002), levels of hemoglobin (HR 0.990, 95% CI 0.981–0.998, *p* = 0.014), and PCT (HR 1.016, 95% CI 1.003–1.029, *p* = 0.014).

**Table 1:** The univariate and multivariate Cox regression analysis for in-hospital mortality.

Variables	Univariate Cox regression analysis			Multivariate Cox regression analysis		
	HR	95% CI	*p* value	HR	95% CI	*p* value
Age	1.045	1.026–1.064	<0.001	1.032	1.011–1.053	0.002
Hypertension	1.165	0.796–1.706	0.432			
Diabetes	1.208	0.771–1.893	0.410			
CKD	2.058	1.261–3.357	0.004	0.991	0.579–1.696	0.974
CHD	0.870	0.523–1.449	0.594			
Heart failure	1.938	1.293–2.905	0.001	1.406	0.919–2.152	0.116
Asthma	0.932	0.295–2.939	0.904			
ILD	2.145	1.145–4.018	0.017	2.036	1.071–3.870	0.030
Cor pulmonale	1.782	1.198–2.651	0.004	1.912	1.276–2.866	0.002
Pulmonary Hypertension	1.252	0.509–3.076	0.625			
Smoke	0.901	0.615–1.322	0.595			
BMI	1.009	0.969–1.050	0.671			
SBP	0.990	0.981–1.000	0.043	1.002	0.991–1.013	0.760
DBP	0.961	0.947–0.976	<0.001	0.971	0.954–0.990	0.002
Pulse	1.008	0.998–1.019	0.113			
HGB	0.982	0.975–0.989	<0.001	0.990	0.981–0.998	0.014
WBC	1.026	0.991–1.062	0.145			
Alb	0.937	0.906–0.969	<0.001	0.988	0.953–1.024	0.505
BUN	1.003	1.000–1.006	0.048	1.001	0.996–1.007	0.599
Creatinine	1.001	1.000–1.003	0.088			
D-Dimer	1.003	0.999–1.008	0.117			
PCT	1.022	1.012–1.031	<0.001	1.016	1.003–1.029	0.014
CRP	1.007	1.005–1.009	<0.001	1.006	1.004–1.008	0.066
ESR	1.005	1.000–1.010	0.053			

CKD, chronic kidney disease; CHD, chronic heart diseases; ILD, interstitial lung disease; BMI, body mass index; SBP, systolic blood pressure; DBP, diastolic blood pressure; HGB, hemoglobin; WBC, white blood cell; BUN, blood urea nitrogen; PCT, procalcitonin; CRP, C reactive protein; ESR, erythrocyte sedimentation rate.

### 3.3 ROC curve and cut-off level of PCT in predicting in-hospital mortality

According to the ROC curve, the continuous PCT level had significant predictive value on in-hospital mortality (AUC: 0.840, 95% CI: 0.802–0.875, *p* < 0.001). An optimal cut-off value of 0.11 ng/mL for PCT was observed in the prediction of in-hospital mortality, as depicted in Figure 2A. This cutoff yielded an area under the curve (AUC) of 83.97%, indicating a high discriminatory capacity. At this threshold, the sensitivity of PCT for detecting in-hospital mortality was 0.844, while the specificity was noted to be 0.717.

**Figure 2:**
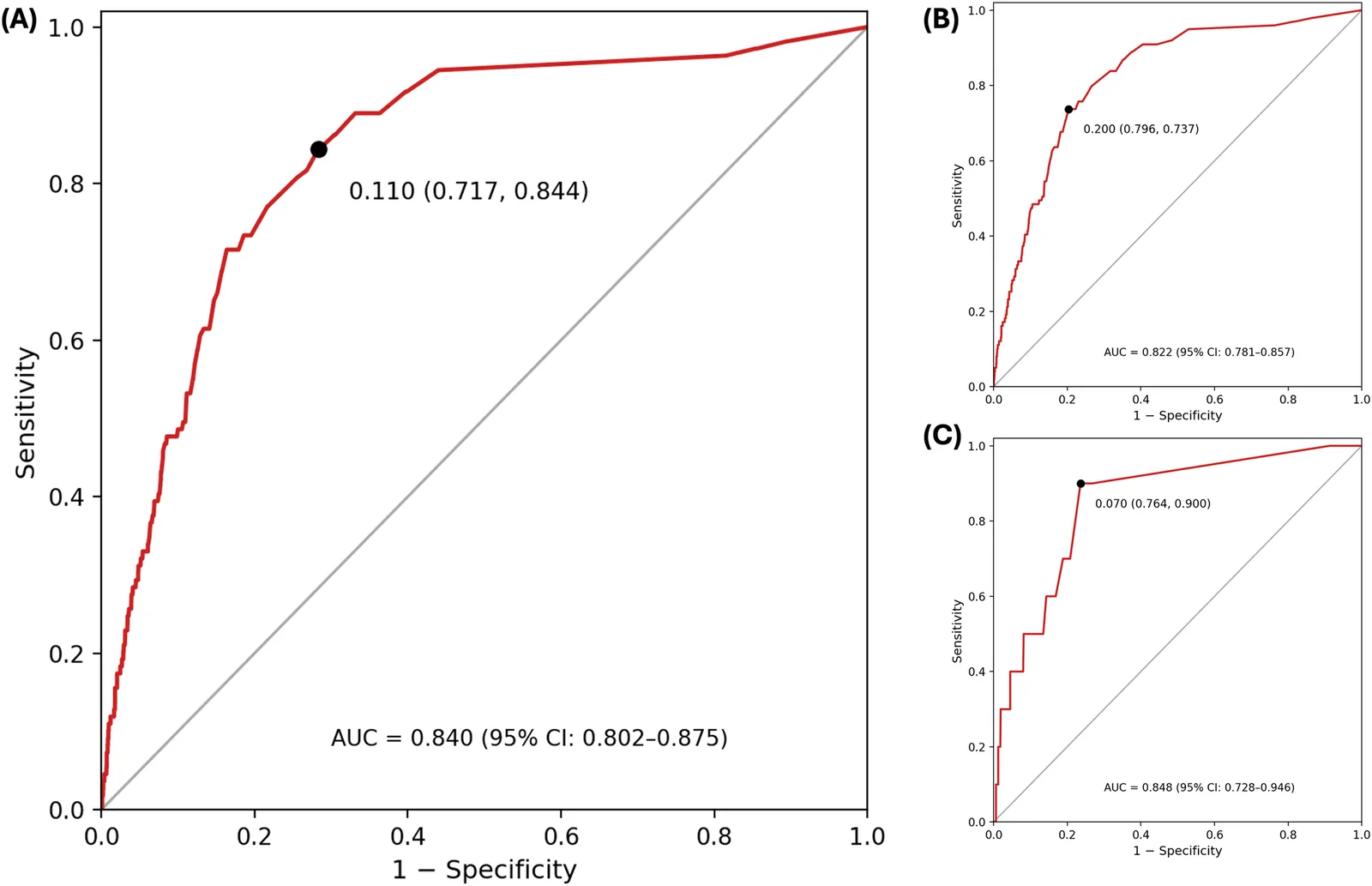
Area under the receiver operating characteristic curve for procalcitonin (PCT) in predicting in-hospital mortality of acute exacerbation of chronic obstructive pulmonary disease (AECOPD): **(A)** in the whole study population; **(B,C)** in the subgroup of patients with/without antibiotic usage during hospitalization.

### 3.4 The baseline characteristics and in-hospital treatment in different PCT groups

Based on the established optimal PCT cutoff value, the study participants were categorized into two groups: the PCT high group, characterized by PCT exceeding 0.11 ng/mL (*n* = 1880), and the PCT low group, where PCT was 0.11 ng/mL or lower (*n* = 4911).

In the PCT high group, as depicted in Table 2, patients were found to be of an advanced age with a reduced BMI. Furthermore, there was a significantly higher incidence of comorbid conditions such as hypertension, coronary heart disease, heart failure, chronic kidney disease, arrhythmia, cor pulmonale, diabetes, and cerebrovascular diseases (all *P* < 0.001) in the PCT high group.

**Table 2:** The characteristics and laboratory tests of the study population in different PCT groups.

Variables	Total (*n* = 6791)	PCT low (*n* = 4911)	PCT high (*n* = 1880)	*p* value
Sex (Female) (%)	1219 (18.0)	881 (17.9)	338 (18.0)	0.998
Age (M, IQR)	72 (65, 79)	71 (64.5, 78)	75 (67, 81)	<0.001
Smoking history (%)	4586 (67.5)	3343 (68.1)	1243 (66.1)	0.131
Hypertension (%)	2371 (34.9)	1655 (33.7)	716 (38.1)	0.001
Coronary heart disease (%)	764 (11.3)	472 (9.6)	292 (15.5)	<0.001
Heart failure (%)	674 (9.9)	365 (7.4)	309 (16.4)	<0.001
CKD (%)	240 (3.5)	74 (1.5)	166 (8.8)	<0.001
Arrhythmia (%)	535 (7.9)	294 (6.0)	241 (12.8)	<0.001
ILD (%)	188 (2.8)	113 (2.3)	75 (4.0)	<0.001
Chronic pulmonary heart disease (%)	1168 (17.2)	767 (15.6)	401 (21.3)	<0.001
Bronchiectasis (%)	563 (8.3)	394 (8.0)	169 (9.0)	0.214
Pulmonary hypertension (%)	185 (2.7)	126 (2.6)	59 (3.1)	0.225
OSAHS (%)	49 (0.7)	38 (0.8)	11 (0.6)	0.508
Diabetes (%)	887 (13.1)	568 (11.6)	319 (17.0)	<0.001
Osteoporosis (%)	169 (2.5)	116 (2.4)	53 (2.8)	0.320
Anxiety or depression (%)	58 (0.9)	41 (0.8)	17 (0.9)	0.896
Cerebrovascular disease (%)	366 (5.4)	233 (4.7)	133 (7.1)	<0.001
Cough (%)	6439 (94.8)	4689 (95.5)	1750 (93.1)	<0.001
Sputum (%)	6328 (93.2)	4614 (94.0)	1714 (91.2)	<0.001
Purulent (%)	1008 (14.8)	664 (13.5)	344 (18.3)	<0.001
Wheezing (%)	1469 (21.6)	1002 (20.4)	467 (24.8)	<0.001
Chest tightness (%)	1355 (20.0)	949 (19.3)	406 (21.6)	0.039
Dyspnea (%)	4292 (63.2)	3194 (65.0)	1098 (58.4)	<0.001
Fever (%)	713 (10.5)	369 (7.5)	344 (18.3)	<0.001
Chest pain (%)	353 (5.2)	242 (4.9)	111 (5.9)	0.119
BMI (kg/m2, M, IQR)	21.23 (18.37, 24.12)	21.26 (18.59, 24.22)	20.90 (17.97, 23.88)	0.002
SBP (mmHg)	131 (120, 145)	132 (120, 145)	129 (117, 144)	<0.001
DBP (mmHg)	78 (70, 86)	78 (70, 86)	76 (68, 84)	<0.001
Pulse (times/min)	89 (80, 101)	88 (80, 100)	91 (80.75, 105)	<0.001
Respiratory rate (times/min)	20 (20, 22)	20 (20, 22)	20 (20, 22)	<0.001
Hb (g/L)	129 (116, 141)	132 (120, 143)	121 (105, 135)	<0.001
WBC (10^9/L)	7.76 (6.00, 10.21)	7.40 (5.82, 9.41)	9.10 (6.74, 12.75)	<0.001
NEUT% (%)	74.1 (64.4, 83.7)	71.3 (62.4, 80.2)	82.2 (72.9, 88.8)	<0.001
Platelet (10^9/L)	200 (151, 257)	202 (157, 258)	193 (139, 252)	<0.001
Respiratory failure (%)	2656 (39.1)	1942 (39.5)	714 (38.0)	0.248
Albumin (g/L)	36.70 (33.50, 39.90)	37.50 (34.50, 40.40)	34.40 (30.40, 37.90)	<0.001
BUN (mmol/L)	5.58 (4.20, 7.50)	5.30 (4.10, 6.90)	6.80 (4.80, 9.83)	<0.001
Creatinine (*μ*mol/L)	77.3 (63.5, 96.0)	76.0 (63.0, 92.3)	81.20 (64.0, 114.0)	<0.001
D-dimer (mg/L)	0.69 (0.38, 1.49)	0.58 (0.33, 1.12)	1.19 (0.59, 2.59)	<0.001
Fibrinogen (g/L)	3.86 (2.98, 5.00)	3.67 (2.90, 4.64)	4.57 (3.31, 6.06)	<0.001
CRP (mg/L)	9.17 (3.07, 32.30)	6.06 (2.26, 17.95)	32.7 (9.53, 97.30)	<0.001
ESR (mm/h)	31 (13, 61)	27 (12, 53)	48 (20, 83)	<0.001
Glucocorticoid using (%)^a^	3245 (47.8)	2324 (47.3)	921 (49.0)	0.229
Antibiotics using (%)^a^	4507 (66.4)	2959 (60.3)	1548 (82.3)	<0.001

PCT, procalcitonin; CKD, chronic kidney disease; ILD, interstitial lung disease; OSAHS, obstructive sleep apnea-hypopnea syndrome; BMI, body mass index; SBP, systolic blood pressure; DBP, diastolic blood pressure; Hb, hemoglobin; WBC, white blood cell; NEUT%, neutrophil percentage; BUN, blood urea nitrogen; CRP, C reactive protein; ESR, erythrocyte sedimentation rate.

a Refers to glucocorticoid/antibiotics using during hospitalization.

As for the spectrum of symptoms characteristic of AECOPD, as depicted in Table 2, cough was the most frequently observed, impacting 94.8% of patients, followed by sputum production at 93.2%, dyspnea at 63.2%, and wheezing at 21.6%. A higher incidence of cough, expectoration, and dyspnea was observed in the PCT low group, while symptoms like purulent expectoration, wheezing, chest tightness, and fever were more pronounced in the PCT high group.

With respect to the initial vital signs, patients in the PCT high group more often had reduced diastolic blood pressure (DBP) and systolic blood pressure (SBP) and increased pulse rate (all *P* < 0.001. As for laboratory tests, patients in PCT high group presented with diminished hemoglobin levels and a lower platelet count, in parallel with a significant increase in the white blood cell (WBC) count and neutrophil percentage (NEUT%) (all *P* < 0.001). Patients with elevated PCT levels also showed markedly higher concentrations of creatinine (CREA), blood urea nitrogen (BUN), D-dimer, fibrinogen, C-reactive protein (CRP), and erythrocyte sedimentation rate (ESR), and a reduced albumin level in the blood (all *P* < 0.001). However, respiratory failure rate was not significantly different (39.5% vs. 38.0%, *p* = 0.248).

Regarding treatment modalities, there was no significant variation in the use of glucocorticoids between the high and low PCT groups during hospitalization (*p* = 0.229). Nonetheless, a higher proportion of patients in the high PCT group were administered antibiotics, with a rate of 82.3% as opposed to 60.3% in the low PCT group (*P* < 0.001).

### 3.5 In-hospital clinical outcomes in different PCT groups

Among the cohort of patients enrolled in our study, the aggregate all-cause mortality rate during hospitalization was recorded at 1.6%, resulting in a total of 109 fatalities as detailed in Table 3. A total of 486 (7.2%) patients were admitted to the ICU. Furthermore, 244 (3.6%) patients underwent invasive mechanical ventilation, and the median LOS in hospital was 8 days (IQR 6–12 days). Notably, patients in the PCT high group exhibited a significantly higher rate of in-hospital mortality, ICU admission, invasive mechanical ventilation, and a prolonged LOS (all *P* < 0.001).

**Table 3:** Clinical outcomes in the study populations.

Variables	Total (*n* = 6791)	PCT low (*n* = 4911)	PCT high (*n* = 1880)	*p* value
In-hospital mortality (%)	109 (1.6)	20 (0.4)	89 (4.7)	<0.001
ICU admission (%)	486 (7.2)	193 (3.9)	293 (15.6)	<0.001
Invasive ventilation (%)	244 (3.6)	72 (1.5)	172 (9.1)	<0.001
LOS (days)	8.00 (6.00, 12.00)	8.00 (6.00, 11.00)	10.00 (7.00, 15.00)	<0.001

PCT, procalcitonin; ICU, intensive care unit; LOS, length of stay.

To further investigate the association between PCT levels and in-hospital mortality, we conducted a Kaplan–Meier survival analysis across varying PCT groups. The findings indicated that patients with elevated PCT levels had a markedly lower survival rate (*P* < 0.0001, Figure 3).

**Figure 3:**
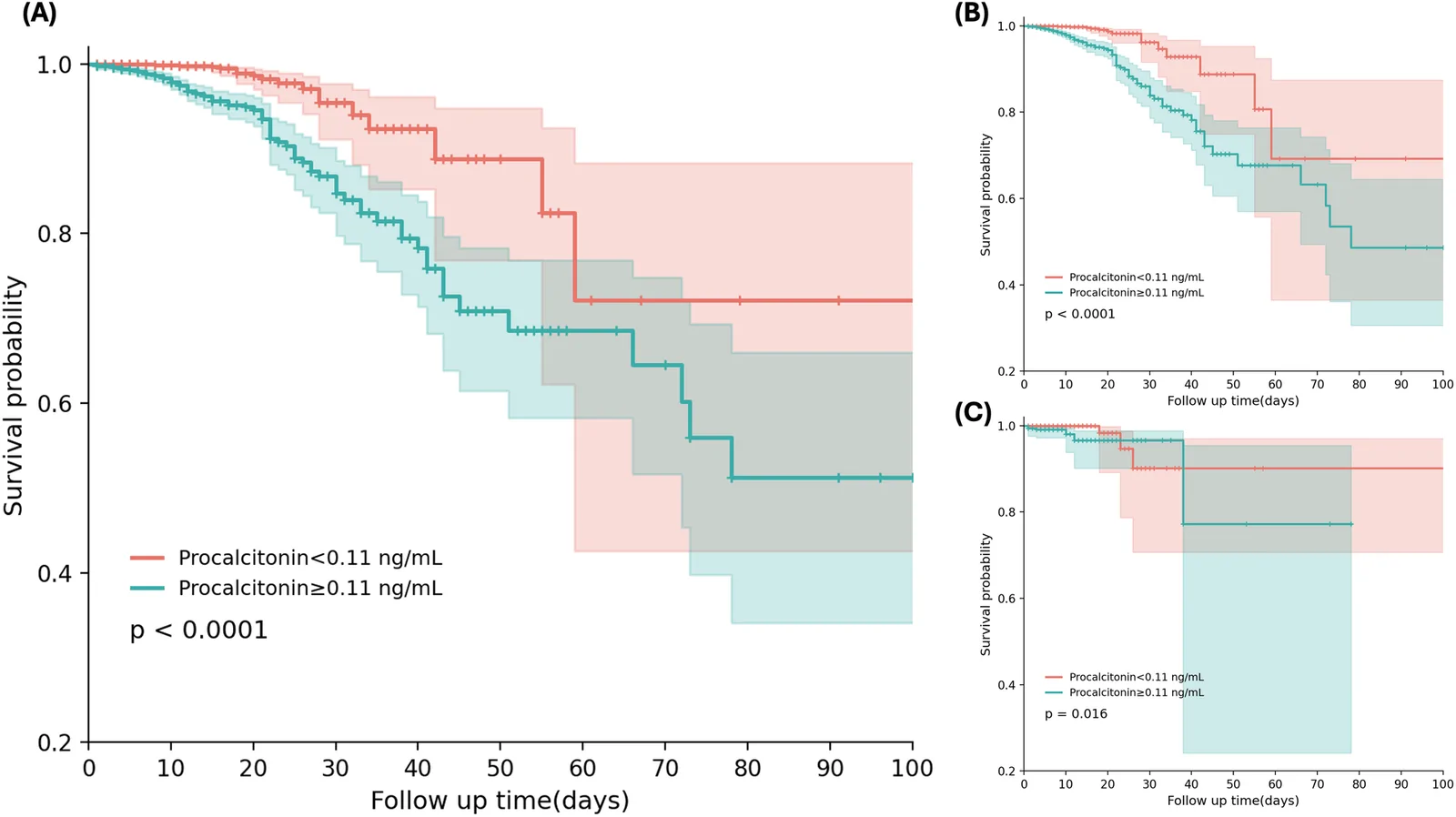
Kaplan–Meier survival analysis of in-hospital mortality in different procalcitonin (PCT) groups: **(A)** in the whole study population; **(B,C)** in the subgroup of patients with/without antibiotic usage during hospitalization.

### 3.6 The subgroup analysis in patients with/without antibiotics

According to the ROC analysis, in patients with/without antibiotics during hospitalization, the continuous PCT value showed good predictive value in both subgroups (with antibiotic: AUC 0.822 (95%CI 0.781–0.857, *p* < 0.001, shown in Figure 2B); without antibiotic: AUC 0.848 (95%CI 0.728–0.946, *p* < 0.001, shown in Figure 2C)). The Youden-derived cutoff were 0.20ng/mL (sensitivity 0.737, specificity 0.796) and 0.07 ng/mL (sensitivity 0.900, specificity 0.764) for patients with or without antibiotics respectively, but the cut-off value of 0.11 ng/mL also showed good predictive efficacy in both subgroups (with antibiotic: AUC 0.753 (95%CI 0.709–0.797, *p* < 0.001); without antibiotic: AUC 0.728 (95%CI 0.547–0.910, *p* = 0.014)). The Kaplan–Meier survival analysis in two subgroups also showed that PCT ≥ 0.11 ng/mL had a markedly lower survival rate in both groups (*P* < 0.05, Figures 3B, C).

### 3.7 The correlation between PCT with clinical laboratory index and aCCI score

To explore the potential link between elevated PCT levels and infection, as well as other markers of inflammation, we conducted a correlation analysis, the results of which are depicted in Figure 4. Contrary to our expectations, we observed only a modest correlation between PCT and CRP, with a correlation coefficient (R) of 0.14. Furthermore, we did not identify any significant associations between PCT levels and a range of other variables, including uric acid, albumin, hemoglobin, platelet count, fibrinogen, ESR, WBC count, Neut, D-dimer, BUN, and CREA.

**Figure 4:**
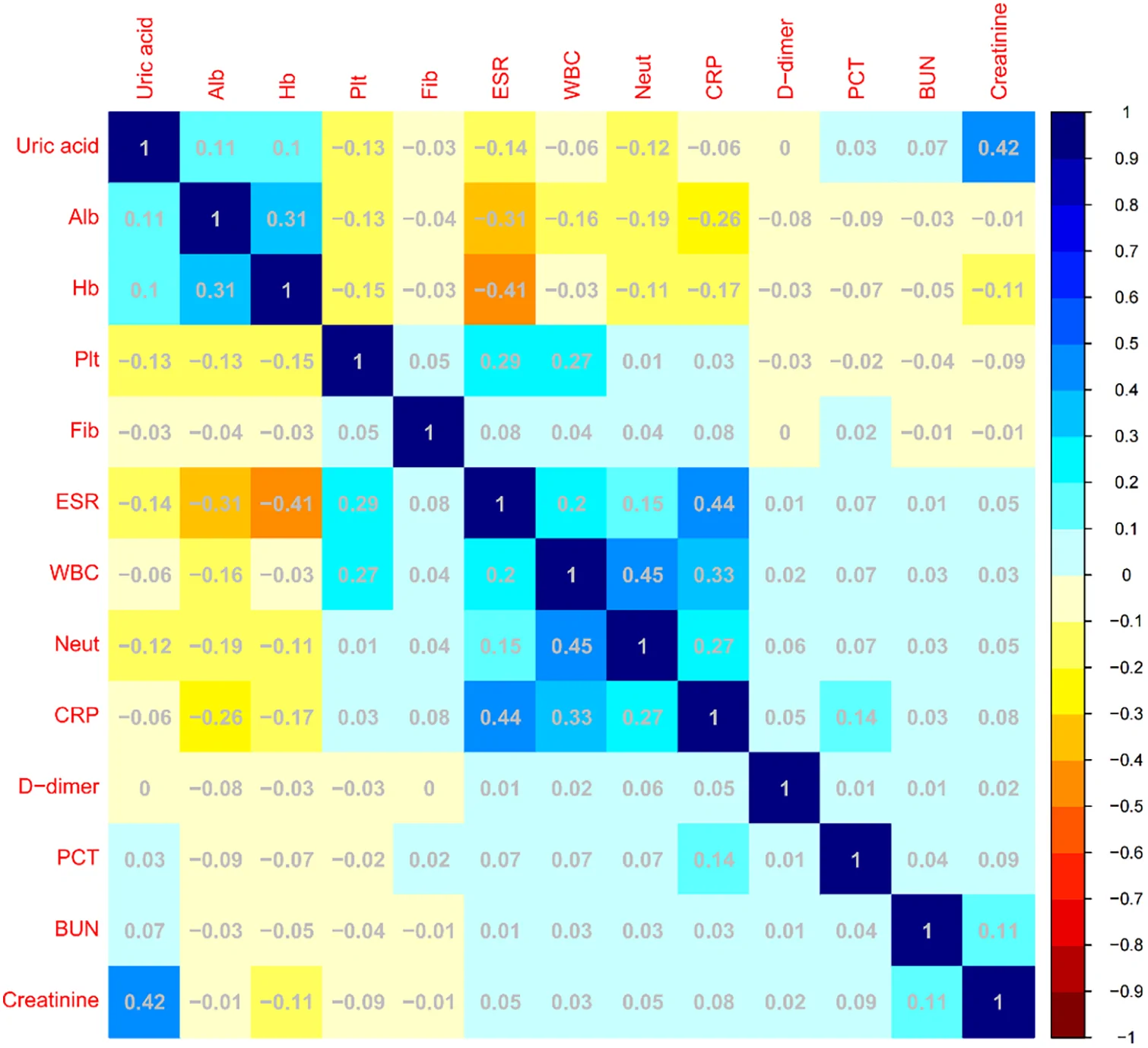
The correlation analysis between procalcitonin (PCT) and clinical laboratory index. Alb, albumin; Hb, hemoglobin; Plt, platelet; Fib, fibrinogen; ESR, erythrocyte sedimentation rate; WBC, white blood cell; Neut, neutrophil; CRP, C reactive protein; BUN, blood urea nitrogen.

To delve into the potential reasons why PCT serves as a predictor of in-hospital mortality in patients with AECOPD, we examined the relationship between PCT levels and the aCCI score. Our analysis revealed that patients with higher PCT levels exhibited relatively higher aCCI score compared to those with lower PCT levels (4.6 versus 4.1, *p* < 0.0001), as illustrated in Supplementary Figure S1.

Subsequently, we conducted a further inquiry into the correlation between PCT and the aCCI score. Interestingly, we discovered a very weak correlation between the two variables (R = 0.032, *p* = 0.0081), as depicted in Supplementary Figure S2.

## 4 Discussion

Within the framework of our prospective, multicenter cohort study, PCT emerged as an independent predictor of in-hospital mortality in AECOPD patients without pneumonia or sepsis, after controlling for age, comorbidities, hemodynamic parameters, and hemoglobin levels. The PCT high group (≥0.11 ng/mL) was characterized by a higher prevalence of comorbid conditions and markedly poorer clinical outcomes. PCT also had significant predictive value of in-hospital mortality in the subgroup of patients with/without antibiotic usage. However, a notable finding was the absence of a strong correlation between PCT levels or other inflammatory biomarkers or the aCCI score.

Respiratory infections are recognized as a prevalent trigger for AECOPD (25), and markedly elevated levels of PCT may suggest a concomitant diagnosis of AECOPD and bacterial pneumonia (14). Nevertheless, PCT elevation was still observed in in AECOPD patients without pneumonia in our study and was significantly associated with adverse prognosis. PCT could further predict the outcome of patients receiving or having not received antibiotics during hospitalization, indicating its prediction value in patients with or without clinical-considered infection. To investigate the reason for PCT elevation in this population, we uncovered the correlation between PCT with aCCI and inflammatory biomarkers. Although patients in the high PCT group exhibited significantly elevated CRP levels and aCCI scores, weak correlations were observed between PCT and these two parameters at the individual level, demonstrating that PCT elevation is not merely a reflection of overall comorbidity burden. The observation does not constitute an inherent contradiction but demonstrate that PCT elevation is not merely a reflection of overall comorbidity burden. It reflects a fundamental distinction between cohort-level epidemiological patterns and individual-level biomarker covariation. The elevated CRP levels and aCCI scores observed at the group level suggest that patients with higher PCT levels tend to bear a heavier burden of systemic inflammation and comorbidities, which was in consistent with previous reports (26, 27). However, the lack of significant linear correlations between PCT and these parameters at the individual level indicates that PCT elevation is neither driven by nor strictly proportional to the degree of CRP or the severity of the comorbidity burden. Instead, PCT appears to capture a distinct, partially independent biological signal, potentially reflecting subclinical bacterial activation, tissue ischemia, or organ stress.

This mechanistic independence between PCT and CRP or aCCI is further substantiated by the finding that PCT remains an independent predictor of in-hospital mortality even after adjusting for age, comorbidities, hemodynamic parameters, and hemoglobin levels. First, the elevated PCT concentration may indicate subclinical or extrapulmonary bacterial infection. Patients with higher PCT showed higher rates of fever (18.3% vs. 7.5%, *P* < 0.001) and purulent sputum (18.3% vs. 13.5%, *P* < 0.001), as well as elevated WBC counts and neutrophil percentages—collectively suggesting a systemic pro-inflammatory and potentially pro-infectious state, even in the absence of confirmed pneumonia. Several mechanisms merit systematic consideration from an infectious disease perspective. As shown in our study, in the PCT high group, significant lower albumin, Hb and BMI were observed. Also, patients with PCT ≥ 0.11 ng/mL had proportion of diabetes, and slightly higher usage of corticosteroid. As a result, these patients are inherently immunocompromised due to corticosteroid use, malnutrition, and impaired immune function (28–30). These factors create susceptibility of these patients to opportunistic and occult infections, including the urinary tract, bloodstream, and gastrointestinal tract. The significantly higher antibiotic utilization in the PCT high group (82.3% vs. 60.3%, *P* < 0.001) further implies that clinicians empirically recognized an infection risk in these patients, even without a formal pneumonia diagnosis. In addition, we discovered the divergence between PCT and CRP in our correlation analysis (R = 0.14) although the median CRP level was much higher in elevated PCT group. CRP is an acute-phase reactant induced by IL-6 in response to a broad range of inflammatory stimuli—both infectious and non-infectious—and consequently lacks specificity for bacterial infection (31). PCT, by contrast, is preferentially induced by bacterial endotoxin (lipopolysaccharide) and by proinflammatory cytokines released in response to systemic bacterial invasion (32). The weak correlation between PCT and CRP in our pneumonia-free AECOPD cohort may therefore reflect the distinct mechanistic pathways underlying their respective elevations: CRP may largely reflect non-specific sterile airway inflammation inherent to AECOPD, while PCT elevation may specifically signal bacterial-driven immune activation—potentially from an extrapulmonary or subclinical source not captured by clinical pneumonia criteria. As a result, it is crucial to closely monitor AECOPD patients with elevated PCT levels, particularly when there is no evidence of bacterial pneumonia.

The predictive value of PCT in the subgroup of patients without antibiotic usage during hospitalization also suggested that PCT could also predict their in-hospital mortality even they were not considered with potential infection. As a result, other than its potential role in signaling occult infection, PCT elevation in non-infectious or mixed clinical states may also reflect systemic tissue stress and proinflammatory cytokine release. PCT has been shown to increase in the context of major surgery, extensive burns, and postoperative ARDS, where bacterial translocation or sterile proinflammatory cascades can stimulate PCT synthesis independently of overt infection (12, 20, 33). Tissue damage in these instances may stimulate the release of proinflammatory cytokines, thus enhancing PCT synthesis (34). In our study, we discovered that in patients with PCT ≥ 0.11 ng/mL, patients had higher proportion of cerebrovascular disease and cardiovascular comorbidities including hypertension, coronary heart disease, chronic pulmonary heart diseases and heart failure. Unexpectedly, either of these comorbidities was recognized as an independent risk factor for in-hospital mortality. The elevated PCT could also be an indicator of higher stress of cardio-/cerebro-vascular system. Additionally, although patients with higher PCT had higher proportion of CKD, it showed scarce association between PCT level and renal function (creatinine, BUN) in our study. Despite impaired renal function, which could affect the clearance of PCT (35, 36), could hardly be the primary driver of PCT elevation in this cohort, high PCT level could also indicate injured status of renal system. Overall, Patients with PCT ≥ 0.11 ng/mL had a higher aCCI score, a longer LOS, and higher rates of in-hospital mortality, ICU admission and invasive mechanic ventilation. The elevated PCT level could indicate a systemic inflammation induced by major tissue injury derived by ischemia, hypoxia, or necrosis. PCT could behave as an acute-phase reactant that can rapidly reflect the overall inflammatory burden and the development of organ dysfunction of patients (37).

From a clinical infectious disease management perspective, our findings carry actionable implications. AECOPD patients presenting with PCT ≥ 0.11 ng/mL in the absence of overt pneumonia constitute a high-risk group that warrants heightened clinical vigilance. We propose that such patients should undergo systematic infection workup at admission—including blood and urine cultures, chest imaging review, and consideration of cultures from other relevant sites—to identify potential sources of occult bacterial infection that may be driving PCT elevation and worsening prognosis. Other than screening the occult infection, these patients require strict monitoring as there could be potential tissue injury inside, which could also drive higher PCT levels. The decision to initiate any treatment therapy should be made in the context of the full clinical picture and the overall status of the patients. Furthermore, this independent factor can be incorporated into existing risk assessment models to enhance prognostic performance and facilitate individualized clinical decision-making in AECOPD inpatients without pneumonia.

To the best of our knowledge, our investigation represents the most extensive study to date regarding PCT as a prognostic marker for outcomes in patients with AECOPD who do not have pneumonia. The present research offers several methodological strengths. Firstly, it is anchored in a nationwide, prospective, multi-center study, ensuring that the sample selection and size are adequately representative. Secondly, PCT, a widely utilized clinical biomarker, is known for its high clinical utility in predicting outcomes for AECOPD patients in real-world settings. However, our study is not without its inherent limitations. This is a non-interventional study, and the treatment protocols were not standardized by the registry protocol, so residual between-center variation in ventilatory management cannot be excluded. Furthermore, although 5997 patients (88.3%) received pulmonary CT imaging in this study, patients with occult radiographic features at admission, or with atypical bacterial pathogens, or with viral pneumonia could also be included. The inclusion of these patients may also influence the findings. Additionally, the study did not account for the possible influence of antibiotics administered prior to hospital admission in some patients, which could potentially skew PCT results. Furthermore, PCT could be potentially tested in sicker patients or those suspected of infection during clinical procedure, so a potential inclusion bias could influence the findings.

## 5 Conclusions

In our study, we discovered that PCT was an independent risk factor for in-hospital mortality in AECOPD patients without pneumonia or sepsis. Individuals with increased PCT levels, exceeding 0.11 ng/mL, were found to have a greater prevalence of comorbidities and a less optimal set of clinical outcomes. The independence of PCT from CRP, renal indices, and overall comorbidity burden suggests that PCT elevation in this population may reflect extrapulmonary or occult bacterial infection, or potential systematic stress or tissue injuries. These findings support PCT as a risk factor in non-pneumonic AECOPD and underscore the need for systematic evaluation in patients with elevated PCT, even in the absence of radiographic pneumonia.

## Data Availability

The raw data supporting the conclusions of this article will be made available from the corresponding author upon reasonable request.

## Supplementary material

Supplementary Figure S1 The age-adjusted Charlson comorbidity index (aCCI) score in different procalcitonin (PCT) groups. *****p* < 0.0001.

Supplementary Figure S2 The correlation between age-adjusted Charlson comorbidity index (aCCI) score and procalcitonin (PCT).
